# DeWitt County Medical Society

**Published:** 1857-09

**Authors:** C. Goodbrake

**Affiliations:** Sec’y


*De Witt County Medical Society.*

The Society met in quarterly session at the school-house in Wapella, July 6, 1857, at 10 o'clock, A. M. Members present —Drs. Adams, McHugh, Lewis, Tyler, Madden, Hunt, Lake, Richards, Wright, Edmiston and Goodbrake; also, Dr. H. Noble and Dr. Laughlin, of Hayworth, McLean county.

The President, Dr. Adams, in the Chair.

The minutes of the previous meeting were read and approved.

On motion of Dr. Lewis, Drs. Noble and Laughlin were invited to participate in the proceedings of the meeting.

The Censors having reported favorable on the application of Joseph C. Ross, M. D., he was, on motion of Dr. Goodbrake, duly elected a member of this Society.

The reading of essays being in order, Dr. Madden read a paper on "Woman and her Diseases," which called forth quite a lengthy discussion, in which most of the gentlemen present partook, pending which the Society adjourned until 2 o'clock, P. M.

AFTERNOON SESSION.

The President called the Society to order at 2 o'clock, P. M.

Dr. McHugh read an essay on "The Practice of Medicine—What it was, what it is, and what it should be." The essay was well written, and was discussed at some length by several of the members present.

Dr. Richards tendered his resignation as Vice President, which was accepted, and Dr. John B. Hunt, of Waynesville, elected to serve as Vice President for the balance of the year.

On motion of Dr. Goodbrake, Dr. Harrison Noble was elected an honorary member of this Society. Dr. Noble, in a very eloquent manner, thanked the Society for the honor conferred.

On motion of Dr. Tyler, the following resolution was unanimously adopted:

*Resolved*, That the thanks of this Society are due and are hereby tendered to Dr. John Wright and lady, for the very excellent and sumptuous dinner served up at their residence for the members of this Society.

On motion, the thanks of this Society were tendered to Drs. Madden and McHugh for their excellent and instructive essays.

The President appointed Dr. B. S. Lewis and Dr. John J. Lake to write each an essay to be read before the Society at its next meeting.

On motion of Dr. Tyler, it was ordered that the proceedings of this meeting be published in the North-Western Medical and Surgical Journal, and the Central Transcript.

On motion, the Society adjourned to meet in semi-annual session at Waynesville, on the first Monday of October next, at 10 o'clock, A. M. C. GOODBRAKE, Sec'y.

